# Evaluation of noninvasive positive pressure ventilation after extubation from moderate positive end-expiratory pressure level in patients undergoing cardiovascular surgery: a prospective observational study

**DOI:** 10.1186/2052-0492-2-5

**Published:** 2014-01-23

**Authors:** Takeshi Suzuki, Takuya Kurazumi, Shinya Toyonaga, Yuya Masuda, Yoshihisa Morita, Junichi Masuda, Shizuko Kosugi, Nobuyuki Katori, Hiroshi Morisaki

**Affiliations:** Department of Anesthesia, Kawasaki Municipal Hospital, 12-1 Shinkawadori, Kawasaki-ku, Kawasaki-shi, Kanagawa 210-0013 Japan; Department of Anesthesiology and General Intensive Care Unit, Keio University School of Medicine, 35 Shinanomachi, Shinjuku-ku, Tokyo 160-8582 Japan

**Keywords:** Weaning, Invasive mechanical ventilation, Noninvasive positive pressure ventilation, Moderate PEEP level, Cardiovascular surgery

## Abstract

**Background:**

It remains to be clarified if the application of noninvasive positive pressure ventilation (NPPV) is effective after extubation in patients with hypoxemic respiratory failure who require the sufficient level of positive end-expiratory pressure (PEEP). This study was aimed at examining the effect and the safety of NPPV application following extubation in patients requiring moderate PEEP level for sufficient oxygenation after cardiovascular surgery.

**Methods:**

With institutional ethic committee approval, the patients ventilated invasively for over 48 h after cardiovascular surgery were enrolled in this study. The patients who failed the first spontaneous breathing trial (SBT) at 5 cmH_2_O of PEEP, but passed the second SBT at 8 cmH_2_O of PEEP, received NPPV immediately after extubation following our weaning protocol. Respiratory parameters (partial pressure of arterial oxygen tension to inspiratory oxygen fraction ratio: P/F ratio, respiratory ratio, and partial pressure of arterial carbon dioxide: PaCO_2_) 2 h after extubation were evaluated with those just before extubation as the primary outcome. The rate of re-intubation, the frequency of respiratory failure and intolerance of NPPV, the duration of NPPV, and the length of intensive care unit (ICU) stay were also recorded.

**Results:**

While 51 postcardiovascular surgery patients were screened, 6 patients who met the criteria received NPPV after extubation. P/F ratio was increased significantly after extubation compared with that before extubation (325 ± 85 versus 245 ± 55 mmHg, *p* < 0.05). The other respiratory parameters did not change significantly. Re-intubation, respiratory failure, and intolerance of NPPV never occurred. The duration of NPPV and the length of ICU stay were 2.7 ± 0.7 (SD) and 7.5 (6 to 10) (interquartile range) days, respectively.

**Conclusions:**

While further investigation should be warranted, NPPV could be applied effectively and safely after extubation in patients requiring the moderate PEEP level after cardiovascular surgery.

## Background

Prolonged invasive mechanical ventilation (IMV) through endotracheal tube, appreciated as the most efficient way to provide sufficient oxygenation in patients with respiratory failure, is ironically likely to cause various complications such as ventilator-induced lung injury and ventilator-associated pneumonia, leading to an increase of mortality [1, 2]. Thus, one of the major goals in the intensive care unit (ICU) is to wean critical ill patients from such IMV as early as possible without consequent respiratory failure and re-intubation. A number of clinical trials have demonstrated that the clinical strategy such as daily interruption of sedation [3], protocol-based sedation [4], and awaking and breathing trial [5] to obviate the prolonged duration of IMV is beneficial.

Previous studies showed that noninvasive positive pressure ventilation (NPPV) is safe and effective to facilitate weaning from IMV in especially medical patients requiring long-term ventilatory support for predominantly hypercapnic respiratory failure [6–8]. However, there are few data which describe the effectiveness of NPPV to facilitate the process of liberation from IMV in patients with hypoxemic respiratory failure. Due to severe postoperative pulmonary complications such as atelectasis, pulmonary edema, pneumonia, and phrenic nerve palsy, postcardiovascular surgery patients often develop hypoxemic respiratory failure requiring high level of positive end-expiratory pressure (PEEP) for sufficient oxygenation and could suffer from prolonged IMV [9, 10]. Although NPPV has been used widely for postoperative respiratory failure [11, 12], there are few studies to examine whether NPPV can be applied effectively and safely after extubation in such population who requires sufficient PEEP level and long-term ventilator support after cardiovascular surgery.

We, therefore, designed the present study to evaluate the effect and the safety of our weaning protocol in which NPPV was applied after extubation in these patients who were mechanically ventilated for over 48 h and simultaneously required moderate PEEP level for sufficient oxygenation after cardiovascular surgery.

## Methods

We conducted this prospective observational study in our eight-bed ICU of Kawasaki Municipal Hospital in Japan. Institutional review board approval was obtained and thereby allowed us to wave the need of informed consent since this is an observational study and the application of NPPV after extubation is performed in a routine manner in our ICU.

### Study protocol

**Figure 1:**
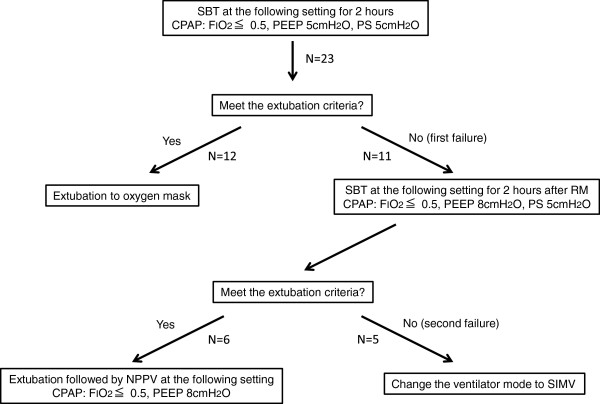
**Study protocol for weaning from invasive ventilator support.** SBT, spontaneous breathing trial; CPAP, continuous positive airway pressure; FIO_2_, inspiratory oxygen fraction; PEEP, positive end-expiratory pressure; PS, pressure support; RM, recruitment maneuver, RM was performed at the pressure of 30 cmH_2_O for 30 s; SIMV, synchronized intermittent mandatory ventilation.

Patients who underwent cardiovascular surgery from April 2011 to December 2011 in our hospital were screened. All patients who received mechanical ventilation via endotracheal tube for over 48 h after cardiovascular surgery were enrolled in this study. Exclusion criteria were younger than 20 years old, body mass index over 35, and chronic renal failure requiring hemodialysis. We excluded patients on hemodialysis since extubation from moderate PEEP level through NPPV application might be hazardous due to the impracticality of constant diuresis in these patients. Additionally, the patients who could not be weaned from IMV or died within 14 postoperative days (POD) were excluded from the study. Eligible patients were screened every morning and considered ready for weaning if they met all the following weaning criteria: (1) hemodynamic stability requiring less than 3 μg/kg/min of dopamine or dobutamine and/or less than 0.02 μg/kg/min of norepinephrine, (2) partial pressure of arterial oxygen tension to inspiratory oxygen fraction ratio (P/F ratio) more than or equal to 200 mmHg with PEEP of 10 cmH_2_O or less, (3) adequate conscious level and cough reflex, and (4) absence of hyperthermia (>38°C) or suspected pneumonia. Pneumonia was suspected in patients with a new or worsened pulmonary infiltrate on chest X-ray who meets any two of the following three criteria: leukocyte count above 12,000 or below 4,000/μL, body temperature above 38°C and/or the presence of purulent respiratory secretions. Patients who met all these weaning criteria were initiated to wean from IMV following our protocol (Figure 1). This protocol was quite different from the conventional one in which the trachea was extubated only after successful spontaneous breathing trial (SBT) at 5 cmH_2_O of PEEP.

The first SBT was performed under continuous positive airway pressure (CPAP) mode with pressure support (PS) for 2 h at the following setting: PEEP equal to 5 cmH_2_O, PS equal to 5 cmH_2_O, and inspiratory oxygen fraction (FIO_2_) equal to or less than 0.5. If the patients could tolerate the first SBT in accordance with our extubation criteria described below, the trachea was extubated. The extubation criteria were as follows: (1) P/F ratio ≧200 mmHg, (2) respiratory ratio <30/min, (3) rapid shallow breathing index (the ratio of respiratory frequency to tidal volume) <105, (4) variability of blood pressure and heart rate <20%, (5) adequate conscious level and cough reflex, and (6) no agitation. If the first SBT failed, the second SBT was performed for 2 h at the following setting immediately after the first SBT followed by recruitment maneuver (30 cmH_2_O for 30 s) application, CPAP mode with PEEP equal to 8 cmH_2_O, PS equal to 5 cmH_2_O, and FIO_2_ equal to or less than 0.5. If the extubation criteria during the second SBT were confirmed, they received sequential NPPV (BiPAP vision, Respironics, Murrysvile, PA, USA) through a full facial mask immediately after extubation. NPPV was delivered at the CPAP mode with 8 cmH_2_O of PEEP and the same FIO_2_ as before extubation. During NPPV, all patients were administered an infusion of dexmedetomidine (0.2 to 0.7 μg/kg/hr) to relieve discomfort. Ventilator setting was not changed for 2 h if O_2_ saturation monitored by pulse oximetry (SpO_2_) was maintained more than 92% and respiratory rate was less than 35 breaths/min. At 2 h after extubation, the setting was adjusted to achieve SpO_2_ >94% and respiratory rate <30 breaths/min if necessary. The physicians in charge were allowed to use S/T mode with different inspiratory and expiratory positive pressure at any time if indicated. NPPV was applied at least for 12 h and weaned by reducing positive pressure level gradually by 2 cmH_2_O while maintaining SpO_2_ >94% and respiratory rate <30 breaths/min. Since the reason why these patients require moderate PEEP for sufficient oxygenation should be overhydration, we dehydrated these patients using diuretics to wean from NPPV while paying strict attentions to hemodynamic stability. Once the PEEP level of 4cmH_2_O and FIO_2_ equal to or less than 0.5 were achieved, arterial blood gases were analyzed 2 h later. If P/F ratio was above 200 mmHg, patients were weaned from NPPV and received O_2_ mask (total flow, 30 L/min; FIO_2_, 0.5). Weaning from NPPV was considered as successful unless respiratory failure developed for 24 h after liberation from NPPV, defined as the presence of any of the following criteria: (1) severe hypoxemia with SpO_2_ <92% or PaO_2_ <70 mmHg at FIO_2_ of 0.5 or more, (2) respiratory rate >35 breaths/min, (3) respiratory acidosis (arterial pH <7.3 with a partial pressure of arterial carbon dioxide (PaCO_2_) >50 mmHg), (4) clinical signs of respiratory muscle fatigue such as use of accessory muscle and paradoxical motion of the abdomen, (5) inability to remove tracheal secretions, or (6) severe dyspnea.

### Criteria for re-intubation

Patients were immediately intubated if any of the following criteria was unmasked: (1) respiratory or cardiac arrest, (2) respiratory pauses with loss of consciousness or gasping, (3) uncontrolled agitation, (4) massive aspiration, (5) the inability to remove respiratory secretions, or (6) hemodynamic instability unresolved by fluids and vasoactive agents. Furthermore, the patients were re-intubated if respiratory failure described above persisted for over 4 h despite adjustment of ventilator setting and possible medical or physical therapy.

### Measurement parameters

At baseline, demographic data, SOFA score at ICU admission, diagnosis for surgery, and duration of IMV before extubation were recorded. Arterial pressure, heart rate, respiratory rate, and SpO_2_ were monitored continuously, whereas arterial blood gases were analyzed at 7 a.m. every morning, just before extubation, 2 h after initiation of NPPV and any time if indicated. The duration of NPPV, the presence of re-intubation or respiratory failure, and the length of ICU stay were also recorded. The primary end-point was the changes of respiratory parameters including P/F ratio, respiratory rate, and PaCO_2_, 2 h after initiation of NPPV compared with those just before extubation. The secondary end-points were the changes of hemodynamic parameters (systolic blood pressure and heart rate) after initiation of NPPV, the rate of re-intubation, the frequency of respiratory failure and intolerance of NPPV, the duration of NPPV, and the length of ICU stay.

### Statistical analysis

Continuous variables were presented as means ± standard deviation (SD), or medians and interquartile ranges, and compared by Student’s *t* test for variables with a normal distribution and by Mann–Whitney U test for variables with a non-normal distribution. Categorial valuables were compared by Fisher’s exact probability test. A *p* value of <0.05 was considered as statistically significant.

## Results

Fifty-one patients underwent cardiovascular surgery during the study period. Among them, 27 patients were weaned from IMV within 48 h after surgery, whereas the rest were screened for the eligibility of this study. One patient was excluded from the study because of chronic renal failure requiring hemodialysis. Of another 23 patients, 12 patients were weaned from IMV to face mask in accordance with the weaning protocol of our institute after the first SBT at 5 cmH_2_O PEEP (Figure 1), of whom 3 patients were re-intubated and 2 of them underwent tracheostomy at 15 and 16 POD. Among other 11 patients, 5 patients did not meet the extubation criteria (second failure). Namely, two patients had tracheostomy at 15 and 21 POD, one patient died at 10 POD due to acute myocardial infarction, and two patients died at 13 and 18 POD due to multiple organ failure with severe sepsis before extubation. After all, six eligible patients who met the extubation criteria after the second SBT were enrolled for this trial. Clinical characteristics of these patients are summarized in Table 1. Before the successful second SBT, five of six patients failed the first SBT because of hypoxemia (P/F ratio <200 mmHg) and another one patient progressed to tachypnea (respiratory rate ≧30 breaths/min).

**Table 1:** **Characteristics of six patients weaned from invasive mechanical ventilation through application of NPPV**

No.	Age	Sex	Height (cm)	Weight (kg)	Diagnosis	Operative procedure	Operation time (h and min)	SOFA score	Duration of IMV (day)	Reason for first failure	Duration of NPPV (day)
1	55	F	161	66	MR, TR	MVR, TAP	7 h and 04 min	8	8	P/F ratio <200 mmHg	2
2	72	M	166	66	Atrial fibrillation	Maze operation	6 h and 00 min	8	3	P/F ratio <200 mmHg	2
3	79	F	148	57	Angina	CABG	5 h and 47 min	7	4	P/F ratio <200 mmHg	4
4	71	M	161	52	Angina	CABG	6 h and 48 min	8	6	RR ≧30/min	2
5	63	M	170	59	Stenosis of right outflow tract	Plasty of right outflow tract	9 h and 47 min	11	8	P/F ratio <200 mmHg	3
6	85	M	160	58	Angina	CABG	3 h and 17 min	5	3	P/F ratio <200 mmHg	3

NPPV, non-invasive positive pressure ventilation; SOFA score, sequential organ failure assessment score; IMV, invasive mechanical ventilation; P/F ratio, partial pressure of arterial oxygen tension to inspiratory oxygen fraction ratio; RR, respiratory ratio; M, male; F, female; MR, mitral regurgitation; TR, tricuspid regurgitation;MVR, mitral valve replacement; TAP, tricuspid annuloplasty; CABG, coronary artery bypass grafting.

In all six patients, NPPV was performed successfully over 12 h without adjusting ventilator setting. The changes of respiratory parameters are described in Table 2. The P/F ratio increased significantly 2 h after initiation of NPPV compared with just before extubation (325 ± 85 mmHg versus 245 ± 55 mmHg, *p* < 0.05), while respiratory rate and PaCO_2_ did not change significantly (17 ± 5.5/min versus 18.2 ± 6.5/min and 33.2 ± 5.3 mmHg versus 34.0 ± 5.1 mmHg, respectively). Hemodynamic parameters including systolic blood pressure and heart rate did not change significantly after initiation of NPPV compared with those before extubation (Table 3). No patients needed re-intubation or developed respiratory failure after application of NPPV. All patients tolerated the whole NPPV procedure. The duration of NPPV was 2.7 ± 0.7 days, and the length of ICU stay was 7.5 (6 to 10) days. However, the rate of re-intubation and the length of ICU stay of these six patients did not reach statistically significant difference compared with those of 12 patients who were extubated to oxygen face mask more than 48 h after surgeries at low PEEP level of 5 cmH_2_O (0/6 versus 3/12; *p* = 0.515, 7.5 (6 to 10) versus 5.5 (4.5 to 8.5) days; *p* = 0.256). All six patients were discharged from the hospital without any severe consequences.

**Table 2:** **The changes of respiratory parameters in six eligible patients before and after NPPV application**

No.	P/F ratio (mmHg)		RR ( /min)		PaCO_2_ (mmHg)	
	Before	After	Before	After	Before	After
1	201.2	287.4	15	12	32.6	33.0
2	248.3	280.4	13	11	33.3	36.5
3	246.4	298.2	16	14	36.7	28.9
4	351.2	458.4	31	25	34.1	34.7
5	206.6	228.6	18	20	41.3	40.6
6	219.2	398	16	20	25.8	25.7
mean ± SD	245 ± 55	325 ± 85*	18.2 ± 6.5	17.0 ± 5.5	34.0 ± 5.1	33.2 ± 5.3

*p < 0.05 compared with before. P/F ratio, partial pressure of arterial oxygen tension to inspiratory oxygen fraction ratio; RR, respiratory ratio; PaCO_2_, partial pressure of arterial carbon dioxide.

**Table 3:** **The changes of hemodynamic parameters in six eligible patients before and after NPPV application**

No.	Systolic BP (mmHg)		HR (beats/min)	
	Before	After	Before	After
1	122	113	89	78
2	145	135	94	87
3	112	121	79	85
4	108	102	82	78
5	138	136	61	65
6	142	128	71	62
mean ± SD	128 ± 16	123 ± 13	79 ± 12	76 ± 10

NPPV, noninvasive positive pressure ventilation; BP, blood pressure; HR, heart rate.

## Discussion

The present study showed that an application of NPPV after extubation is safe and effective in patients requiring moderate level of PEEP after cardiovascular surgery. In particular, P/F ratio at 2 h after induction of NPPV improved significantly rather than before extubation. Furthermore, all patients weaned from NPPV successfully without the need of re-intubation and the development of respiratory failure.

NPPV has been used widely now not only in ICU but also in general wards [13], and the efficacy of this technique has been reported in various clinical situations, such as exacerbation of chronic obstructive pulmonary disease (COPD) [14], cardiac pulmonary edema [15], and postoperative respiratory failure [11, 12]. Although some previous studies examined the effect of NPPV to facilitate a weaning from IMV in patients who required long-term ventilator support and suffered from persistent weaning failure [6–8], our study is quite different from these previous studies in some points. Contrary to previous studies which included medial ICU patients with predominantly hypercapnic COPD patients, our study targeted only patients who have risk factors for developing hypoxemic respiratory failure after cardiovascular surgery. Besides, we applied NPPV after extubation in patients who could maintain sufficient oxygenation at PEEP level of 8 cmH_2_O but failed at 5 cmH_2_O PEEP following our weaning protocol in this study. We guessed that the major reasons why these six patients required moderate PEEP level were atelectasis, overhydration, and cardiogenic pulmonary edema due to the long procedure of cardiac surgery and large amount of transfusion required for perioperative hemodynamic stability. Thus, it is unclear whether this weaning technique can be generalized to other type of hypoxemic respiratory failure patients. However, one recent small randomized controlled study, including 20 patients, demonstrated that NPPV application after early extubation from moderate level of PEEP was beneficial in non-surgical patients with resolving hypoxemic respiratory failure [16]. Furthermore, early application of nasal CPAP after extubation from 7 cm H_2_O PEEP was reported to reduce pulmonary morbidity and length of hospital stay following the surgical repair of thoracoabdominal aortic aneurysms [17]. Thus, weaning strategy through NPPV for patients requiring moderate level of PEEP might be beneficial in wide range of hypoxemic respiratory failure, which should be confirmed by a large randomized controlled trial.

Oxygenation improved significantly after the initiation of NPPV compared with before extubation even though the applied PEEP level was the same (8 cmH_2_O). While the exact mechanisms for this improvement is unknown, some possible mechanisms are considered, such as absence of sedation other than dexmedetomidine, increased patient’s activity, and improvement of dorsal ventilation leading to reduced V/Q mismatch. Although this improvement of oxygenation could support the use of this weaning strategy, there are some concerns to apply this technique. Delayed intubation after developing respiratory failure is associated with worse outcome [18, 19], thereby re-intubation should not be hesitated if respiratory failure once develops. The protocol to prevent delayed re-intubation should be made in all ICU where patients with respiratory failure are treated by NPPV. Tolerance of patients is also one of the key factors in managing NPPV successfully. Although patients were sedated with remifentanil or propofol to increase NPPV tolerance in previous studies [20, 21], we used continuous dexmedetomidine infusion, which has little respiratory depressant effects, to relieve discomfort as a routine practice, probably contributing to the high successful rate of NPPV management in our ICU.

There are several limitations to interpret the data herein. *First*, this study is not a randomized controlled trial but an observational study, and the sample size was too small. It remains unknown whether this technique can reduce the length of mechanical ventilation, the rate of complication relating to IMV, the length of ICU stay, and the mortality compared with the conventional weaning strategy. However, all 6 patients enrolled in this study could wean from IMV through application of NPPV without any progression to respiratory failure and the need of re-intubation, while 3 of 12 patients who were liberated from IMV to oxygen face mask were re-intubated even though they passed SBT at the low PEEP level (5 cmH_2_O). Although the rate of re-intubation did not reach significant difference, this might be attributed to the small sample size. Whether this weaning strategy could reduce the rate of re-intubation, it should be evaluated in a randomized controlled trial in the future. *Second*, we performed two sequential SBT for 4 h in this study protocol which could be too long procedure, while some weaning guideline recommends SBT should be performed every 24 h [22]. However, there are no evidences as to the appropriate duration of SBT. *Finally*, there is no rationale for 8 cm H_2_O of PEEP level at which trachea were extubated in this study. It may be plausible that patients could be liberated from IMV at higher level of PEEP, which should be examined in a future study.

## Conclusions

Application of NPPV after liberation from IMV via tracheal intubation was safe and effective in patients who required moderate level of PEEP for sufficient oxygenation after cardiovascular surgery. The P/F ratio improved significantly 2 h after initiation of NPPV compared with that just before extubation, and all patients could wean successfully without development of respiratory failure and re-intubation. A randomized controlled trial is warranted to confirm the effectiveness of this technique before it is widely used in other ICU.

## Abbreviations

COPD: chronic obstructive pulmonary disease
CPAP: continuous positive airway pressure
FIO_2_: inspiratory oxygen fraction
ICU: intensive care unit
IMV: invasive mechanical ventilation
NPPV: noninvasive positive pressure ventilation
P/F ratio: PaO_2_ to FIO_2_ ratio
PaCO_2_: partial pressure of arterial carbon dioxide
PaO_2_: partial pressure of arterial oxygen tension
PEEP: positive end-expiratory pressure
PS: pressure support
SBT: spontaneous breathing trial
SD: standard deviation
SOFA score: sequential organ failure assessment score
SpO_2_: O_2_ saturation monitored by pulse oximetry
